# G-protein-coupled receptors mediate 14-3-3 signal transduction

**DOI:** 10.1038/sigtrans.2016.18

**Published:** 2016-09-30

**Authors:** Hua Li, Alex Eishingdrelo, Sathapana Kongsamut, Haifeng Eishingdrelo

**Affiliations:** 1 BioInvenu Corp., East Hanover, New Jersey, USA; 2 Rudder Serendip LLC, Madison, New Jersey, USA

## Abstract

G-protein-coupled receptor (GPCR)-interacting proteins likely participate in regulating GPCR signaling by eliciting specific signal transduction cascades, inducing cross-talk with other pathways, and fine tuning the signal. However, except for G-proteins and β-arrestins, other GPCR-interacting proteins are poorly characterized. 14-3-3 proteins are signal adaptors, and their participation in GPCR signaling is not well understood or recognized. Here we demonstrate that GPCR-mediated 14-3-3 signaling is ligand-regulated and is likely to be a more general phenomenon than suggested by the previous reports of 14-3-3 involvement with a few GPCRs. For the first time, we can pharmacologically characterize GPCR/14-3-3 signaling. We have shown that GPCR-mediated 14-3-3 signaling is phosphorylation-dependent, and that the GPCR/14-3-3 interaction likely occurs later than receptor desensitization and internalization. GPCR-mediated 14-3-3 signaling can be β-arrestin-independent, and individual agonists can have different potencies on 14-3-3 and β-arrestin signaling. GPCRs can also mediate the interaction between 14-3-3 and Raf-1. Our work opens up a new broad realm of previously unappreciated GPCR signal transduction. Linking GPCRs to 14-3-3 signal transduction creates the potential for the development of new research directions and provides a new signaling pathway for drug discovery.

## Introduction

G-protein-coupled receptor (GPCR) signal transduction is pluridimensional. Upon agonist stimulation, GPCRs activate heterotrimeric G-proteins, leading to the dissociation of G-proteins and the generation of second messengers. G-protein-coupled receptor kinases (GRKs) then phosphorylate activated receptors, leading phosphorylated receptors to recruit signal adaptors or cellular effectors. Interactions between GPCRs and an array of signal adaptors or cellular effectors modulate both G-protein-dependent and G-protein-independent signaling pathways, and offer the possibility of eliciting specific spatiotemporal signaling cascades, inducing cross-talk with other signaling pathways, and fine tuning and specifically regulating GPCR signaling at multiple levels.^1,2^

In addition to well-documented G-protein-dependent and β-arrestin-dependent GPCR signaling pathways, other cellular effectors are recruited to GPCRs. One of them is the multifunctional signal adaptor protein 14-3-3. The first evidence of 14-3-3 as a GPCR cellular effector was demonstrated with the α_2_-adrenergic receptors (α_2_ARs).^3^ Binding of 14-3-3 to the third intracellular loop of α_2_ARs regulates receptor cellular localization and coordinates signal transduction. Interaction of 14-3-3 proteins with other GPCRs such as thromboxane receptors,^4^ parathyroid hormone receptor,^5^ cannabinoid receptor^6^ and others^7–9^ has been associated with various cellular functions. 14-3-3 has been found to interact with the C-terminal domain of GABA_B1_ and to inhibit the heterodimerization of GABA_B1_ and GABA_B2_ at their C-terminal domains.^10^ Proteomic analysis has also indicated an association between 14-3-3 and angiotensin II type 1a receptor (AT1aR).^11^ Recently, bioinformatic analysis has predicted that 68% of neurotransmitter GPCRs have 14-3-3-binding motifs.^12^

14-3-3 proteins are ~30 kDa proteins that are ubiquitously expressed in eukaryotic cells but that are most highly expressed in the brain, where they make up ~1% of total soluble protein. Although they lack enzymatic activity, 14-3-3 proteins bring two or more proteins together to form signal complexes.^13^ The remarkable feature of 14-3-3 proteins is the number and diversity of their protein interaction partners. Bioinformatic and proteomic analyses predict that >2000 phosphoproteins interact with 14-3-3 proteins.^14^ Their interaction partners include kinases, phosphatases, ubiquitin ligases, transcription factors, scaffold proteins, cytoskeletal proteins and membrane proteins, including GPCRs, receptor tyrosine kinases, cytokine receptors and ion channels.^15^ These interactions facilitate the formation of large molecular complexes that coordinate the responses of multiple signaling pathways to incoming stimuli, allow signal transduction among different cellular compartments and carry out a variety of physiological functions. Not surprisingly, 14-3-3 proteins are also associated with human diseases, most notably with cancers and with neurological disorders such as Alzheimer’s disease, Parkinson’s disease, spinocerebellar ataxia type 1, schizophrenia and bipolar disorder, based on the evidence from both clinical and laboratory studies.^16,17,18,19^

Although evidence shows that 14-3-3 forms complexes with some GPCRs, investigation of GPCR-mediated 14-3-3 signaling has been largely ignored. The inability to assess specific 14-3-3 signaling is a major reason for such studies to lag behind studies of G-protein and β-arrestin signaling pathways. Significant progress in pharmacologically characterizing GPCR-mediated 14-3-3 signaling depends on the availability of research tools and methods. Here we show that, like β-arrestins, 14-3-3 proteins interact with activated GPCRs to form signaling complexes that mediate signal transduction. By applying LinkLight assay technology^20,21^ to assess GPCR/14-3-3 protein interactions, we can pharmacologically characterize GPCR-mediated 14-3-3 signaling pathways.

We show for the first time that several GPCRs signal through 14-3-3, and that GPCR antagonists can specifically block agonist-induced 14-3-3 signaling. And, we find out that GPCR-mediated 14-3-3 signaling is phosphorylation-dependent. The pan-kinase inhibitor staurosporine blocks GPCR-mediated 14-3-3 signaling, whereas the GRK2/3 inhibitor CMPD101 does not. Moreover, we demonstrate that the GPCR/14-3-3 interaction occurs on a slower timescale than the GPCR/β-arrestin interaction. In addition, using the β_3_AR, which lacks a β-arrestin interaction motif, as an example, we demonstrate that GPCR-mediated 14-3-3 signaling can be β-arrestin-independent. Furthermore, we compare assays of GPCR-mediated 14-3-3 and β-arrestin signaling pathways by profiling a panel of agonists. The data indicate that a ligand can exert different activity in different signaling pathways. Finally, we investigated the interaction between 14-3-3 and the signal effector Raf-1. The Raf-1/14-3-3 interaction can be stimulated by serum and GPCR agonists through endogenous receptors, and this interaction is blocked by staurosporine. Linking GPCR signaling to the vast 14-3-3 protein interaction networks provides the potential for understanding GPCR signaling at a more complex level, as well as for the development of new approaches that target specific protein–protein interactions that may aid GPCR drug discovery.

## Materials and methods

### Materials

Compounds and chemicals were purchased from Sigma-Aldrich (St Louis, MO, USA) and Tocris Biosciences (Bristol, UK).

### Cell lines and cell culture

HEK293 cells were routinely maintained and passaged in standard Dulbecco’s modified Eagle medium (DMEM) with 10% fetal bovine serum (FBS) and penicillin/streptomycin (Gibco, Grand Island, NY, USA, catalog # 15070). U2OS cells were purchased from the American Type Culture Collection (ATCC, Manassas, VA, USA) and were cultured with McCoy’s 5A medium (Gibco catalog # 16600-082) supplemented with 10% FBS (Gibco catalog # 26140-079) and 1× penicillin/streptomycin (Gibco catalog # 15070). Cells were cultured in a 37 °C incubator with 5% CO_2_. Cell culture medium was replaced every 3–4 days, and cells were passaged at 90% confluence.

### Plasmid construction and generation of stable cell lines

Full-length complementary DNAs of human GPCRs without stop codons were subcloned in frame with the tobacco etch virus (TEV) protease vector as previously described.^20,21^ The β-arrestin-2-permuted luciferase (β-arr-2-pLuc) expression plasmid was also previously described.^20,21^ 14-3-3ɛ and 14-3-3ζ full-length complementary DNAs without stop codons were used to replace β-arrestin-2 in the β-arr-2-pLuc for construction of the 14-3-3ɛ-pLuc and 14-3-3ζ-pLuc expression plasmids. Transfections of HEK293 and U2OS cells were performed with Lipofectamine 2000 transfection reagent according to the manufacturer’s instructions (Life Technologies, Carlsbad, CA, USA, catalog # 11668-027). Monoclonal cell lines stably expressing both GPCR-TEV and the 14-3-3ɛ-pLuc fusion protein were selected using 400 μg ml^−1^ G418 (Invitrogen, Grand Island, NY, USA/Life Technologies, catalog # 10131-027) and 100 μg ml^−1^ Hygromycin B (Life Technologies, catalog # 10687-010). Multiple stable clones were selected for evaluation.

### Luciferase assay

The LinkLight cells stably expressing both GPCR-TEV and 14-3-3ε-pLuc were seeded into a 384-well white plate (Becton Dickinson, Franklin Lakes, NJ, USA) at 20 000 cells per well with 30 μl of regular medium. Cells were cultured overnight. The following day, the culture medium was replaced with 15 μl of serum-free DMEM, followed by the addition of 5 μl of compounds/drugs. Agonists were added as serial dilutions in serum-free DMEM from 10 mm dimethylsulfoxide stocks. For antagonist inhibition assays, the medium was replaced with 10 μl of serum-free DMEM. Antagonists or inhibitors were added at 5 μl per well and incubated with the cells for 15 min. Then, the stimulant or agonist was added at 5 μl per well and incubated for 240 min or for other times as indicated. An equal volume of Bright-Glo (Promega, Madison, WI, USA), One-Glo (Promega), BriteLite (PerkinElmer, Billerica, MA, USA) or NeoLite (PerkinElmer) luciferase detection reagent was added for a 5-min incubation. The luminescence signals were determined using a luminescence plate reader (EnSpire or TopCount). Alternatively, after compound incubation, the culture medium was replaced with 10 μl of luciferase detection reagent, and luminescence signals were recorded.

### Measurement of the effects of agonists and antagonists on GPCR/14-3-3 signaling

The GPCR/14-3-3 LinkLight cells were seeded in white 384-well plates. After overnight culture, the culture medium was replaced with serum-free DMEM, and a serial dilution of GPCR ligand was added to the cells. After agonist incubation (240 min unless otherwise specified), a luciferase detection reagent was added to the cells, and luminescence signals were recorded. The antagonist effects were measured after the cells were preincubated with the compounds for 15 min, followed by the stimulation with 1 μm agonists for 240 min before proceeding to the luciferase assay.

### Measurement of agonist-induced GPCR/β-arrestin-2 signaling

The GPCR/β-arrestin-2 LinkLight cells were seeded in white 384-well plates. After overnight culture, the culture medium was replaced with serum-free DMEM, and a serial dilution of GPCR ligand was added to the cells. After agonist incubation (120 min unless otherwise specified), a luciferase detection reagent was added to the cells, and luminescence signals were recorded.

### Measurement of GPCR agonist-induced Raf-1/14-3-3ε interaction

The Raf-1/14-3-3ε LinkLight cells were seeded in white 384-well plates. After overnight culture, the culture medium was replaced with 15 μl of serum-free DMEM/ per well, and a serial 1:3 dilution of adrenaline, dopamine or 5'-N-ethylcarboxamidoadenosine (NECA) (5 μl per well) was added to the cells. After 180 min of ligand incubation, a luciferase detection reagent was added to the cells, and luminescence signals were recorded.

### Measurement of FBS-stimulated Raf-1/14-3-3ε interaction

The Raf-1/14-3-3ε LinkLight cells were seeded in white 384-well plates. After overnight culture, the culture medium was replaced with 15 μl of serum-free DMEM per well, and a serial 1:2 dilution of FBS (5 μl per well) was added to the cells. After 180 min of FBS incubation, a luciferase detection reagent was added to the cells, and luminescence signals were recorded.

### Measurement of kinase inhibitor responses

The LinkLight cells were cultured in a white 384-well plate overnight. The culture medium was then replaced with serum-free DMEM. Serial dilutions of kinase inhibitors were added to cells for 15 min, followed by agonist stimulation for 240 min and then by the luciferase assay as described above. For Raf-1/14-3-3 kinase inhibition assays, cells were preincubated with a kinase inhibitor (or two inhibitors at equal concentrations) for 15 min, followed by stimulation with 7.5% FBS for 180 min and then by the luciferase assay as described above.

### Data analysis

Concentration–response curves were analyzed using Prism software (GraphPad Software, Inc., San Diego, CA, USA). All values are expressed as mean±s.d. (*n*=3).

## Results

### Development of a GPCR/14-3-3 LinkLight assay

**Figure 1:**
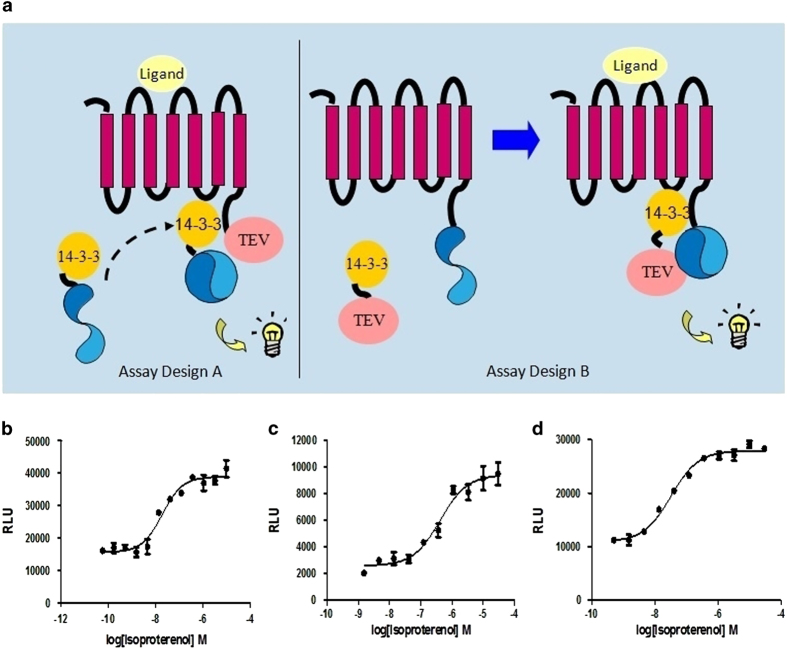
Development of the GPCR-mediated 14-3-3 signaling assay. (**a**) A schematic drawing of design A for the GPCR/14-3-3 signaling LinkLight assay. Agonist-activated GPCR-Tobacco Etch Virus protease (TEV) interacts with a pLuc-tagged 14-3-3 protein. The close proximity between the TEV-tagged GPCR and the pLuc-tagged 14-3-3 leads to proteolytic cleavage of the permuted luciferase (pLuc). The cleaved luciferase fragments spontaneously refold to reconstitute an active luciferase, driven by the high affinity of fragment self-complementation. The luminescence signals are then detected after the addition of luciferase detection reagent. Right: a schematic drawing of design B for the GPCR/14-3-3 signaling LinkLight assay. The alternative design B switches tags between GPCR and 14-3-3, with the GPCR tagged with pLuc and 14-3-3 tagged with TEV. (**b**) Concentration–response curve for the ADRB2-TEV/14-3-3ɛ-pLuc LinkLight assay. The experiment was performed as a proof of concept of design A by transiently expressing ADRB2-TEV and 14-3-3ɛ-pLuc in cells, followed by the treatment with serial dilutions of the ADRB2 agonist isoproterenol. (**c**) Concentration–response curve for the ADRB2-TEV/14-3-3ζ-pLuc LinkLight assay. The 14-3-3ζ isoform was used as an alternative, to verify the GPCR/14-3-3 interaction concept. (**d**) Concentration–response curve of for the ADRB2-pLuc/14-3-3ɛ-TEV LinkLight assay (design B). The experiment was performed as a proof of concept of design B by transiently expressing ADRB2-pLuc and 14-3-3ɛ-TEV in cells, followed by the treatment with serial dilutions of the ADRB2 agonist isoproterenol. RLUs, relative light units.

Although there have been several studies showing that 14-3-3 proteins participate in GPCR signaling, pharmacologically characterizing them has not been easy. Because 14-3-3 proteins have no intrinsic enzymatic activity, an approach to measure protein interaction ability is needed. Previously, we developed GPCR/β-arrestin and ERK2/β-arrestin interaction LinkLight assays.^20,21^ These assays respectively assess GPCR-mediated β-arrestin signaling and ERK2/ β-arrestin interaction resulting in ERK2 signaling. Similarly, to develop GPCR/14-3-3 interaction LinkLight assays (Figure 1a), we can utilize the interaction between the GPCR and 14-3-3 as the readout for GPCR-mediated 14-3-3 signaling. An inactive permuted luciferase (pLuc) is created by breaking luciferase into two fragments, rearranging the fragment order in that the original N-terminal fragment is moved to the C terminus and the original C-terminal fragment is moved to the N terminus, and reconnecting them using a TEV protease cleavage sequence. We can attach pLuc to the C terminus of 14-3-3 (design A) and TEV protease to the C terminus of GPCR. Upon the formation of GPCR/14-3-3 signal complex, TEV protease is close to pLuc, resulting in cleavage of inactive pLuc. The cleaved luciferase fragments are spontaneously refolded, driven by fragment self-complementation affinity, and active luciferase is reconstituted. Alternatively, the tags can be switched between interaction partners (design B).

We used the β_2_-adrenergic receptor (ADRB2) as a prototype for developing GPCR/14-3-3 signaling assays. In humans, there are seven 14-3-3 proteins; all the isoforms contain the K49, R56 and R127 residues that are critical for the binding of 14-3-3 to different partners.^22^ We selected 14-3-3 epsilon (ε) and 14-3-3 zeta (ζ) for the assays, and we constructed 14-3-3ε-pLuc and 14-3-3ζ-pLuc expression plasmids. We transiently expressed ADRB2-TEV with 14-3-3ε-pLuc or ADRB2-TEV with 14-3-3ζ-pLuc in HEK293 cells, and we then looked for luciferase signals induced by stimulation with the ADRB2 agonist isoproterenol. Cells transfected with either construct produced concentration-dependent signals in response to isoproterenol, indicating that agonist-activated ADRB2 interacted with 14-3-3 proteins. To further demonstrate ligand-induced formation of ADRB2/14-3-3 signal complexes, we also tested assay design B (reversing TEV and pLuc) by co-expressing ADRB2-pLuc and 14-3-3ε-TEV in HEK293 cells. The result of assay design B confirmed ADRB2/14-3-3 protein interaction in an agonist-dependent manner (Figures 1b–d). Now, for the first time, we can pharmacologically characterize the GPCR-14-3-3 interaction.

### Application of GPCR/14-3-3 LinkLight assay to additional GPCRs

**Figure 2:**
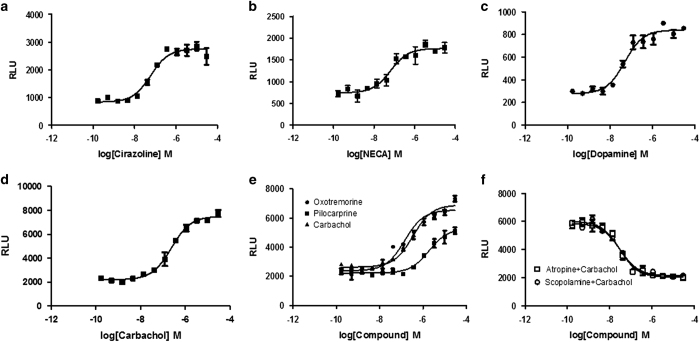
Concentration–response curves of GPCR-mediated 14-3-3 signaling with various GPCRs. (**a**) Concentration–response curve of α1A-TEV/14-3-3ɛ-pLuc stably transfected cells treated with the α1A agonist cirazoline. (**b**) Concentration–response curve of ADORA2A-TEV/14-3-3ɛ-pLuc stably transfected cells treated with the adenosine receptor agonist NECA. (**c**) Concentration–response curve of DRD2-TEV/14-3-3ɛ-pLuc stably transfected cells treated with dopamine. (**d**) Concentration–response curve of CHRM3-TEV/14-3-3ɛ-pLuc stably transfected cells treated with the muscarinic acetylcholine receptor agonist carbachol. (**e**) Concentration–response curve of CHRM5-TEV/14-3-3ɛ-pLuc stably transfected cells treated with a panel of muscarinic acetylcholine receptor agonists: oxotremorine, pilocarpine and carbachol. (**f**) Concentration–response curves of CHRM5-TEV/14-3-3ɛ-pLuc stably transfected cells treated with the muscarinic acetylcholine receptor antagonists scopolamine and atropine and then stimulated with the agonist carbachol (3 μm). RLUs, relative light units.

Although a number of GPCRs, including cannabinoid receptor type 1, κ-Opioid Receptor, thromboxane receptors, parathyroid hormone receptor, angiotensin II type 1 receptor and orphan GPR15, have been reported to interact with 14-3-3 proteins, the pharmacology and physiology of GPCR-mediated 14-3-3 signaling is poorly understood. Multiple 14-3-3 protein-binding motifs have been characterized, and putative binding sites on GPCRs can be predicted,^12^ although 14-3-3-binding sites in numerous proteins do not conform to these optimal motifs. 14-3-3 proteins can bind to phosphorylated motifs that differ from the consensus and can even bind to unphosphorylated motifs, presumably because other structural features also contribute to the interactions.^23^ Therefore, we selected a few more GPCRs to investigate their 14-3-3 signaling pathways. We generated multiple HEK293 cell lines stably co-expressing GPCR-TEV and 14-3-3ε-pLuc, and examined agonist-induced GPCR-mediated 14-3-3 signaling. Alpha adrenergic receptor 1A (α1AR), adenosine receptor A2a (ADORA2A), dopamine receptor D2 (DRD2), muscarinic cholinergic receptor 3 (CHRM3) and 5 (CHRM5) all showed agonist-stimulated 14-3-3 signaling (Figures 2a–e). The assay EC_50_ values for the respective agonists of α1AR (cirazoline, 9.5 nm), DRD2 (dopamine, 12 nm), CHRM3 (carbachol, 21 nm), and CHRM5 (carbachol, 24 nm) are comparable to the reported values from G-protein signaling assays. We treated stable CHRM5/14-3-3 LinkLight cells with the agonists oxotremorine, pilocarpine and carbachol to produce concentration-dependent 14-3-3 signals. Oxotremorine and carbachol are full agonists, and pilocarpine behaves as a partial agonist for 14-3-3 signaling. Next, we investigated whether antagonists could inhibit agonist-induced GPCR-mediated 14-3-3 signals. Preincubation of the cells with various concentrations of antagonists (scopolamine or atropine) followed by treatment with 3 μm carbachol produced antagonist concentration-dependent inhibition signals (Figure 2f).

### Time course of GPCR-mediated 14-3-3 signaling

**Figure 3:**
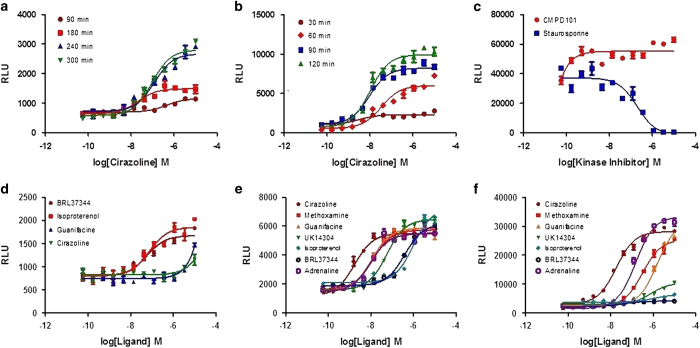
Time course and phosphorylation dependence of GPCR-mediated 14-3-3 signaling. (**a**) Concentration–response curves of α1A-TEV/14-3-3ɛ-pLuc stably transfected cells treated with the α1A agonist cirazoline for various times, as shown. (**b**) Concentration–response curves of α1A-TEV/β-arrestin-pLuc stably transfected cells treated with the α1A agonist cirazoline for various times. (**c**) Concentration–response curves for the kinase inhibitors CMPD101, H-89 and staurosporine blocking signaling in α1A-TEV/14-3-3ɛ-pLuc stably transfected cells. (**d**) Concentration–response curves of a panel of adrenergic receptor agonists on ADRB3-TEV/14-3-3ɛ-pLuc stably transfected cells. (**e**) Concentration–response curves of a panel of adrenergic receptor agonists on α1A-TEV/14-3-3ɛ-pLuc stably transfected cells. (**f**) Concentration–response curves of a panel of adrenergic receptor agonists on α1A-TEV/β-arrestin-pLuc stably transfected cells. RLUs, relative light units.

Agonist-occupied GPCRs activate G-proteins in a matter of a few seconds, whereas arrestin-bound GPCRs exist in a relatively stable complex that persists on a timescale of minutes to hours.^24,25^ The time course of the interaction between GPCR and 14-3-3 is unknown. The LinkLight assay cannot assess the kinetics of protein–protein interactions, but we can compare the time needed to reach maximum signals between the GPCR/14-3-3 interaction and the GPCR/β-arrestin interaction. We thus examined the relative time courses of achieving maximum signals for GPCR-mediated 14-3-3 and β-arrestin signaling pathways. We treated α1AR/14-3-3 and α1AR/β-arrestin cells with agonists for various lengths of time and compared the relative time courses of the β-arrestin and 14-3-3 signaling pathways (Figures 3a and b). For the α1AR-mediated activated β-arrestin signaling pathway, we observed significant signals within 60 min of agonist treatment, the maximum signal is achieved between 120 and 180 min of agonist treatment, and signals start to decrease at 240 min (Figure 3b). However, for the α1AR-mediated 14-3-3 signaling pathway, we observed barely any signal after 90 min of agonist treatment. The signals reached a plateau at ~240 min, indicating that the interaction of 14-3-3 with the GPCR occurs at a later time than the GPCR/β-arrestin interaction (Figure 3a).

### Phosphorylation-dependent GPCR/14-3-3 interaction

GRKs and cyclic AMP-dependent protein kinase (PKA) phosphorylate agonist-occupied receptors on serine or threonine residues within the C terminus or the third intracellular loop.^26^ Similarly, protein interactions with 14-3-3 are largely regulated by the phosphorylation state of the binding partners. 14-3-3-binding motifs usually contain phosphorylated serine or threonine residues.^27^ Therefore, we investigated whether GPCR-activated 14-3-3 signaling could be blocked by a GRK2 and GRK3 (GRK2/3) inhibitor, CMPD101, or a PKA inhibitor, H-89. Preincubation of α1AR/14-3-3 cells with CMPD101 or H-89 did not inhibit signals stimulated by the agonist cirazoline, suggesting that GRK2/3 and PKA do not contribute to promoting the α1AR/14-3-3 interaction. We then used the pan-kinase inhibitor staurosporine in a similar experiment. Pretreatment of α1AR/14-3-3 cells with staurosporine (IC_50_: 19.7 nm) blocked signals stimulated by the agonist cirazoline (3 μm). These data indicate that the GPCR/14-3-3 interaction is phosphorylation-dependent, and that one or more kinases participate in the GPCR-mediated 14-3-3 signaling pathway (Figure 3c).

### β-Arrestin-independent GPCR-mediated 14-3-3 signaling

Does the GPCR/14-3-3 interaction depend on the GPCR/β-arrestin interaction? We used the β_3_ adrenergic receptor (ADRB3) as an example to investigate this question. ADRB3 lacks consensus sequences for GRK phosphorylation and therefore does not recruit β-arrestins, and it does not display short-term agonist-induced functional desensitization or sequestration.^28^ We generated HEK293 cells stably expressing ADRB3-TEV and 14-3-3-pLuc fusion proteins and assessed a panel of adrenergic agonists for their ability to promote an interaction between ADRB3 and 14-3-3. The ADRB3 agonist BRL37344 and the βAR agonist isoproterenol promoted ADRB3/14-3-3 interaction signals in a concentration-dependent manner, whereas the α1AR agonist cirazoline and the α_2_AR agonist guanfacine showed very weak signals even at 10 μm (Figure 3d). These data suggest that GPCR-mediated 14-3-3 signaling can be β-arrestin-independent.

### Profiling agonist-activated GPCRs in 14-3-3 versus β-arrestin signaling

Having demonstrated that GPCRs interact with 14-3-3, we wanted to compare the GPCR-mediated 14-3-3 and GPCR-mediated β-arrestin signaling pathways. We used α1AR as an example. A panel of adrenergic agonists, including the α1AR agonists cirazoline and methoxamine, the α_2_AR agonists guanfacine and UK14304, the β-adrenoceptor agonist isoproterenol, the ADRB3 agonist BRL37344, and the pan-adrenoceptor agonist adrenaline, was used for profiling the activity of these receptors in inducing 14-3-3 versus β-arrestin signaling (Figures 3e and f). The rank order of potency for the 14-3-3 signaling assay is cirazoline (1.72 nm)>adrenaline (9.6 nm)>methoxamine (11.1 nm)>guanfacine (12.4 nm)>UK14304 (55.2 nm)>BRL37344 (386 nm)>isoproterenol (995 nm). The rank order of potency for those agonists in β-arrestin signaling is cirazoline (18.4 nm)>adrenaline (152 nm)>methoxamine (318 nm)>UK14304 (780 nm)>guanfacine (1610 nm). BRL37344 and isoproterenol showed much lower signal strength and behaved as much weaker agonists of β-arrestin signaling than of 14-3-3 signaling. These data suggest that individual ligands may not exert equal potency on different signaling pathways.

### Interaction of 14-3-3 with the signaling effector Raf-1

**Figure 4:**
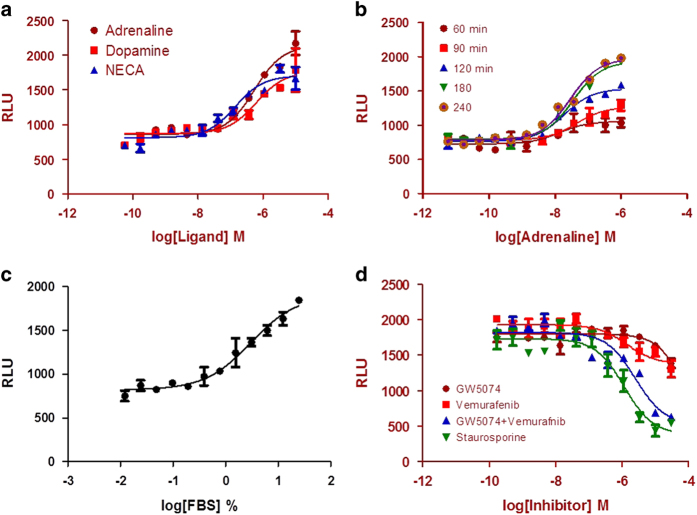
Interaction of 14-3-3 with a downstream effector, Raf-1. (**a**) Stimulation of the Raf-1/14-3-3 interaction by GPCR ligands: adrenaline, dopamine and NECA. (**b**)Time course of the Raf-1/14-3-3 interaction signals in response to adrenaline stimulation. (**c**) Concentration–response curve for FBS. (**d**) Inhibition of the FBS-stimulated Raf-1/14-3-3 interaction by kinase inhibitors: GW5074, vemurafenib and staurosporine. RLUs, relative light units.

We have thus far described the GPCR side of the interaction with 14-3-3. We also performed experiments to examine the signaling effector side of the interaction with 14-3-3. One of the many signaling effectors that bind to 14-3-3 proteins is Raf-1. Activation of GPCRs can lead to activation of the Ras-dependent Raf/MEK/ERK signaling pathway.^29^ Activated Ras recruits Raf-1 to the plasma membrane, where it becomes activated and in turn activates the Raf/MEK/ERK pathway. Binding of 14-3-3 is required for the maintenance of Raf-1 phosphorylation and kinase activity.^30^ Thus, we can utilize Raf-1/14-3-3 protein interaction signals as an indicator of Raf-1 activation. U2OS cells contain several endogenous GPCRs, including adrenergic, dopamine and adenosine receptors. Previously, we showed GPCR agonist-induced ERK2/β-arrestin-2 interaction signals in U2OS cells.^21^ We generated U2OS cells stably co-expressing Raf-1-TEV and 14-3-3ɛ-pLuc, and used those cells to examine the Raf-1/14-3-3 interaction in response to the treatment with GPCR agonists. Stimulation of the cells with adrenaline, dopamine or NECA produced concentration-dependent Raf-1/14-3-3 interaction signals (Figure 4a). The time course of the Raf-1/14-3-3 assay indicates that maximum signals are reached between 120 and 180 min with adrenaline treatment (Figure 4b). Activation of receptor tyrosine kinases is a major stimulus for activating the Raf/MEK/ERK signaling pathway. Treatment of the cells with a serial dilution of FBS produced FBS concentration-dependent Raf-1/14-3-3 interaction signals (Figure 4c). Presumably, growth factors in the serum activate endogenous receptor tyrosine kinases, promoting the interaction between Raf-1 and 14-3-3. Because Raf phosphorylation and kinase activity are regulated by interaction with 14-3-3,^30^ we examined whether kinase inhibitors could block the Raf-1/14-3-3 interaction signals. The pan-kinase inhibitor staurosporine blocked FBS-stimulated Raf-1/14-3-3 interaction signals. At high concentrations, staurosporine suppressed the Raf-1/14-3-3 signals below the baseline signal, suggesting that there is a basal level of Raf-1/14-3-3 interaction under our cell culture conditions. We also treated cells with the Raf-1 kinase inhibitor GW5074 and the BRAF kinase inhibitor vemurafenib. Separately, GW5074 showed very weak inhibition activity, and vemurafenib showed partial inhibition of Raf-1/14-3-3 interaction signals. However, the combination of these two inhibitors enhanced the inhibition of the interaction signals (Figure 4d).

## Discussion

Signal transduction depends on protein–protein interactions. Numerous and diverse phosphoproteins have been found to display binding affinity for 14-3-3 proteins.^14^ These interactions are usually transient, regulated by phosphorylation and dephosphorylation, and turned off once a signal has been transduced to a downstream effector. Thus, 14-3-3 proteins serve as a signal network ‘hub,’ dynamically interacting with their partner proteins and modulating diverse cellular processes. It is not surprising that everything is connected, but how GPCR-mediated 14-3-3 signaling links to the vast 14-3-3 protein interaction networks and thus modulates the interconnected signal transduction networks remains to be explored. It is conceivable that perturbation at one node could affect entire signal networks and that compensation, cross-talk, redundancy and feedback mechanisms eventually calibrate the signal networks to regulate cellular processes and functions in a spatiotemporal manner. Here we have demonstrated that GPCR-mediated 14-3-3 signaling is likely to be a more general phenomenon than has previously been shown for a few GPCRs. However, how widespread the GPCR/14-3-3 signaling pathway turns out to be will need to be experimentally mapped out. Bioinformatic analysis suggests that there are numerous 14-3-3-binding sites both on the GPCR side and on the effector side.^12,14^ Demonstration of GPCRs stimulating/modulating 14-3-3 signaling pathways adds yet another layer to the already-complicated process of GPCR signal regulation. Because 14-3-3 proteins interact with such a large and diverse array of proteins, dissecting the specific functionality of GPCR-mediated 14-3-3 signaling may have to rely on the identification of biased ligands that selectively modulate GPCR-mediated 14-3-3 signaling. Identification of ligands biased towards 14-3-3 signaling would be another major endeavor.

All seven closely related 14-3-3 isoforms contain the K49, R56 and R127 residues that are critical for the binding of 14-3-3 to various partners.^22^ All except 14-3-3σ have been shown biochemically to interact with GPCRs. Activation of cannabinoid receptor type 1 promotes 14-3-3β binding and induces 14-3-3β-mediated signaling.^6^ 14-3-3ζ binds three subtypes of α_2_ARs but binds each with a different potency.^3^ Agonist-mediated β1AR/14-3-3ε binding regulates the hERG potassium channel Kv11.1. 14-3-3η binds the C-terminal tail of parathyroid hormone receptor and can contribute to the regulation of parathyroid hormone receptor signaling.^5^ 14-3-3τ has been found to interact with follicle stimulating hormone receptor.^8^ 14-3-3θ binds the class C GPCR calcium-sensing receptor and attenuates receptor-mediated Rho kinase signaling.^31^ A comparative structural analysis shows that the 14-3-3 proteins are structurally adaptable, and their internal flexibility is likely to facilitate recognition and binding of their interaction partners.^32^ The cell-type-specific expression, subcellular distribution and availability of 14-3-3 isoforms may determine which 14-3-3 isoforms participate in GPCR-mediated signaling. It is likely that different GPCRs may have different binding affinity to different 14-3-3 isoforms, and a specific signaling pathway may be determined by the involvement of a particular 14-3-3 isoform. This situation may resemble GPCR/β-arrestin interactions. Both β-arrestin-1 and β-arrestin-2 interact with GPCRs, and functional differences may depend on the abilities of the two β-arrestins to interact with different proteins and transduce various signals.^33^ Our results showing that ADRB2 interacts with 14-3-3ε and 14-3-3ζ indicate that GPCRs can interact with multiple 14-3-3 isoforms.

Signal traffic analysis in terms of who, when and where may help us to understand signal transduction networks. We compared the GPCR/14-3-3 and GPCR/β-arrestin interactions for relative timescale in terms of reaching maximum signals with our LinkLight assays. We found that the GPCR/14-3-3 interaction takes place over a longer time than the GPCR/β-arrestin interaction. Presumably, GPCRs have been desensitized or internalized and phosphorylated when 14-3-3 proteins come to bind to GPCRs. Does the GPCR/14-3-3 interaction require the participation of β-arrestin? To address this question, we used β_3_AR, which does not recruit β-arrestins upon agonist stimulation. We demonstrated that β_3_AR interacts with 14-3-3 upon on β-adrenoceptor agonist stimulation, suggesting that GPCR-mediated 14-3-3 signaling can be β-arrestin-independent, at least in this case. GPCR signaling is extremely complex, and our understanding of its underlying mechanisms and biological outputs is far from complete.

We have demonstrated that GPCR-mediated 14-3-3 signaling is phosphorylation-dependent. GRKs phosphorylate agonist-occupied GPCRs at serine and threonine residues to create β-arrestin binding sites and to direct internalization through clathrin-coated pits. PKA also participates in GPCR phosphorylation and directs internalization via a caveolar pathway. Similarly, phosphorylated serine and threonine residues are also included in 14-3-3-binding motifs. This prompted us to test whether the GRK2/3 inhibitor CMPD101 and the PKA inhibitor H-89 could block GPCR-mediated 14-3-3 signaling. We found that CMPD101 and H-89 did not block GPCR-mediated 14-3-3 signaling, whereas the pan-kinase inhibitor staurosporine blocked GPCR-mediated signaling, suggesting that another kinase or kinases may participate in the GPCR phosphorylation that creates 14-3-3 protein-binding sites.

In addition to the agonist-promoted GPCR/14-3-3 protein interaction, we also investigated whether Raf-1 interacts with 14-3-3. Ras, in response to receptor activation, switches from its inactive (GDP-bound) form to its active (GTP-bound) form, and it then recruits Raf to the membrane and promotes Raf dimerization and activation. Raf-1 kinase activity is known to require 14-3-3 protein binding.^29^ GPCRs can lead to activation of the Raf/MEK/ERK pathway.^30^ We showed that Raf-1/14-3-3 interaction signals were stimulated by the GPCR agonists adrenaline, dopamine and NECA, presumably through endogenous GPCRs, in U2OS cells. The time course of the Raf-1/14-3-3 signals indicates that the Raf-1/14-3-3 interaction occurs before the GPCR/14-3-3 interaction. That result also indicates that 14-3-3 proteins participate in GPCR signal transduction at multiple levels; first, participating in the GPCR-activated Raf/MEK/ERK pathway, and, second, directly interacting with GPCRs. FBS also promoted the interaction signals; presumably, growth factors in the serum bind to endogenous tyrosine kinase receptors and activate the Ras/Raf MAPK pathway. That binding depends on phosphorylation because the pan-kinase inhibitor staurosporine blocked Raf-1/14-3-3 signals. The Raf-1 kinase inhibitor GW5074 showed little activity and the BRAF kinase inhibitor vemurafenib showed partial activity for blocking Raf-1/14-3-3-association signals. However, in combination, they significantly blocked the Raf-1/14-3-3 protein interaction. Raf activation requires Raf homodimerization or heterodimerization (Raf-1/BRAF heterodimer). Therefore, it is likely that simultaneously inhibiting Raf-1 and BRAF blocked Raf-1/14-3-3 interaction signals. Potentially, the Raf/14-3-3 LinkLight assay could be used for identifying new Raf inhibitors or modulators. Activation of the Raf/MEK/ERK signaling pathway can be either Ras-dependent or Ras-independent.^34^ To identify which pathway is utilized for the Raf-1/14-3-3 interaction, a Ras/Raf-1 direct interaction assay needs to be developed. Such an assay could also be a useful tool for identifying blockers or modulators of Ras/Raf-1 interactions.

Historically, GPCR drug discovery research has relied on G-protein signaling pathways for assessing the activity of various compounds. The discovery of G-protein-independent and β-arrestin-dependent signaling pathways created an opportunity for identifying pathway-selective or signaling-biased ligands.^35^ These biased ligands may maximize clinical effectiveness and minimize unwanted side effects. The identification of signaling pathway-dependent ligands challenged the early conventional views of GPCR activation and suggested the existence of multiple conformations induced by different ligands.^36^ Conceptually, a ligand may bind and stabilize a receptor in a conformation that may exert differential activity on the 14-3-3 signaling pathway in addition to the G-protein and β-arrestin signaling pathways. We profiled the effects on the α1A receptor of a panel of adrenergic agonists in both the 14-3-3 and β-arrestin signaling pathway assays. We found that the rank of order of potency, as well as the absolute potency, of these agonists differs between the two different signaling pathway assays, suggesting that a single ligand could possess different activity on different pathways. Signaling pathway-biased ligands that may have differential activity for 14-3-3, G-protein and β-arrestin signaling pathways have yet to be identified. Such biased ligands would produce unequal activation of specific signaling pathways and would produce distinct physiological outcomes. Using the new technique described here, a high-throughput screening campaign to identify ligands biased towards 14-3-3 signaling is now possible. Demonstration that GPCR-mediated 14-3-3 signaling can be directly measured, and that we can pharmacologically characterize ligands modulating this pathway opens up a new realm of previously unappreciated GPCR signal transduction. Consequently, the GPCR-mediated 14-3-3 signaling pathway provides the potential for understanding GPCR signaling pathways more deeply, as well as for the development of novel strategies that target specific GPCR intracellular signaling pathways.
